# One size does not fit all: rethinking IO–TKI use in favorable-risk metastatic ccRCC

**DOI:** 10.3389/fonc.2026.1783114

**Published:** 2026-02-23

**Authors:** Alejandro Valdés, Enrique Grande, Mauricio Burotto

**Affiliations:** 1 Bradford Hill Clinical Research Center, Santiago, Chile; 2 Medical Oncology Department, Instituto Nacional del Cancer, Santiago, Chile; 3 Facultad de Medicina, Universidad Francisco de Vitoria, Pozuelo de Alarcón, Spain; 4 Medical Oncology Department, Hospital Universitario Quironsalud Madrid, Madrid, Spain; 5 Universidad Finis Terrae, Santiago, Chile

**Keywords:** clear cell renal carcinoma, favorable risk, first-line treatment, IMDC, immunotherapy, IO, renal cell carcinoma, TKI

## Introduction

The current standard of treatment for advanced clear cell renal cell carcinoma (ccRCC) is based on immuno-oncology (IO) drugs with immune checkpoint inhibitors (ICI) in combination (IO + IO) or with a VEGF tyrosine-kinase inhibitor (VEGF-TKI; IO + TKI) (1, 2). Treatment is guided according to the International Metastatic RCC Database Consortium (IMDC), a prognostic model developed in the pre-immunotherapy era (3, 4). In patients with IMDC favorable-risk disease (0 points), who account for approximately 20% of advanced ccRCC cases, current guidelines recommend IO + TKI as the preferred first line treatment, with nivolumab + ipilimumab (IO + IO) recently included as a preferred regimen in the NCCN guidelines (5–7). A subset of favorable risk patients have an indolent biology translated into an asymptomatic disease with metachronous lung-limited or glandular (e.g. pancreas or thyroid) metastases and long intervals between nephrectomy and recurrence (8–10). While IO + TKI has emerged as a near-default first-line option in favorable-risk ccRCC, its unselective use risks overtreatment, avoidable toxicity, and inefficient resource utilization, underscoring the need for a more biology-driven, patient-centred approach.

## Discussion

In different molecular subgroup analysis, favorable-risk patients are consistently enriched for an angiogenic molecular phenotype, with high expression of VEGF-related angiogenic signatures, an immune-cold microenvironment and frequent *PBRM1* mutations (11–15). These tumours have good responses to VEGF-TKIs and may rely less on immune-mediated mechanisms for control (16). IMDC risk groups and transcriptomic subtypes are overlapping but non-identical, with some favorable-risk patients exhibiting prolonged responses to ICIs.

IO + TKI regimens have consistently improved objective response rates (ORR), median progression-free survival (mPFS) and median overall survival (mOS) over sunitinib in unselected or intermediate/poor-risk populations in phase III trials (17). However, an efficacy–survival discordance emerges when we focus on the IMDC favorable-risk subgroup (**Table 1**).

**Table 1:** Summary of first-line for advanced ccRCC IO + IO or IO + TKI phase III trials in favorable-risk patients (19,38–57).

Trial	Checkmate 214	Keynote-426	Checkmate 9ER	Clear (Study 307)	JAVELIN renal 101
Intervention versus comparator	Nivolumab + ipilimumab followed by nivolumab versus Sunitinib	Pembrolizumab + Axitinib versus Sunitinib	Nivolumab + Cabozantinib versus Sunitinib	Lenvatinib + Pembrolizumab (L + P) versus Lenvatinib + Everolimus (L+ E) versus Sunitinib (S) (1:1:1)	Avelumab + Axitinib versus Sunitinib
Total number of patients (Number and % of favourable risk)	1096 patients (249 patients, 23%).	861 patients (269 patients, 31.2%).	651 patients (146 patients, 22%).	1069 patients (348 patients, 32%).	886 patients (196 patients, 22%).
ORR Favorable Risk	ORR 29.6% versus 51.6% (CR 12.8% versus 6.5%).	ORR 68.8% versus 50.4% (CR 13% versus 6.1%).	ORR 67.6% versus 45.8% (CR 16.2% versus 9.7%).	L + P versus S: ORR 68.2% versus 50.8% (CR 20.9% versus 4.8%).	ORR 75.5% versus 48.8% (CR 9.6% versus 5.2%).
mPFS Favorable Risk	12.4 versus 28.9 months (HR 1.76, 95 % CI 1.25-2.48)	20.7 versus 17.9 months (HR 0.76, 95% CI 0.57-1.02)	21.4 versus 13.9 months (HR 0.72, 95% CI 0.49-1.05)	L + P versus S: 28.6 versus 12.9 months (HR 0.50, 95% CI 0.35-0.71)	20.7 versus 13.8 months (HR 0.75, 95% CI 0.53-1.07; p=0.1109)
OS Favorable Risk	77.9 versus 66.7 months (HR 0.82 95 % CI 0.60-1.13)	60.3 versus 62.4 months (HR 1.10 95% CI 0.79-1.54)	NR versus 47.6 months (HR 1.07 95% CI 0.63-1.79)	L + P versus S: NR versus 59.9 months (HR 0.94 95% CI 0.58-1.52)	mOS 79.4 versus 65.5 months (HR 0.73, 95% CI 0.48-1.10; p=0.129)

While ORR, complete response (CR) and mPFS with IO + TKI are higher than with sunitinib in a favorable-risk scenario, no combination has demonstrated a statistically significant improvement in overall survival (OS) over sunitinib. Moreover, meta-analyses with longer follow up did not show any significant OS benefit either, but trials were underpowered for this subgroup (18). Upfront IO + TKI is preferred in symptomatic disease, bulky tumor burden, or anatomically threatening sites where rapid cytoreduction is needed. The use of ICIs offers the possibility of long-term control in some patients, with concerns about missing a therapeutic window if ICIs are deferred. In the CheckMate 214 trial (**Table 1**), IMDC favorable-risk patients had inferior PFS and ORR with nivolumab plus ipilimumab (IO + IO) compared to TKI but updated results show better CRs and mOS in the long term (19). Patients with low disease burden who value durable disease control and potential treatment-free intervals may be particularly suitable candidates for an IO+IO combination, recognizing its lower early response probability.

With extended follow-up, the OS benefit observed with IO + TKI regimens often diminishes, with the Cox Hazard Ratio (HR) attenuating toward unity as additional events accrue. This likely reflects non-proportional hazards, whereby IO + TKI provides substantial early disease control, but the relative hazards converge over time owing to treatment discontinuation, emergence of resistance, and effective post-progression therapies in the comparator arm. In contrast, nivolumab plus ipilimumab (IO+IO) may exhibit a delayed, “tail-driven” benefit: in the IMDC favorable-risk subgroup, early analyses numerically favored sunitinib (OS HR 1.45), whereas longer follow-up shows an improving HR with a more evident late survival plateau, consistent with durable benefit in a subset of patients.

In favorable-risk patients with TKI monotherapy (usually first-line sunitinib or pazopanib), mOS has consistently ranged from 4 to 5 years, with a substantial survival tail (20, 21). TKIs produce high rates of grade ≥3 adverse events, but most are predictable and titratable: hypertension, diarrhoea, hand–foot syndrome, fatigue and cytopenias (22). Dose and schedule individualisation, proactive toxicity management, and intermittent treatment strategies (such as those tested in the intermittent treatment STAR trial) can make long-term TKI therapy surprisingly compatible with a good quality of life (23–25). In this context, IO + TKI is not the only “standard”; it is one of several evidence-based options. For many favourable-risk patients, the more conservative options—active surveillance (AS) or TKI alone—may be better aligned with their biology, comorbidities, and life priorities (26–28).

IO + TKI adds toxicity rather than swapping it, making toxicity one of the strongest arguments against universal IO + TKI. ICIs have fewer chronic, low-grade symptoms but more severe immune-mediated adverse events, often early and sometimes irreversible (endocrinopathies). Grade ≥3 adverse events of any cause often exceed 70%, with all the chronic, cumulative TKI toxicities, plus superimposed immune-mediated events and higher rates of treatment discontinuation for toxicity (29, 30). In a fit patient with bulky disease, that trade-off may be acceptable if it delivers a clear survival and symptom benefit. In an asymptomatic patient, subjecting them to years of combination toxicity without proven OS gain looks far less attractive.

To date, no IO + TKI combination has demonstrated a quality-of-life (QoL) benefit in the IMDC favorable-risk subgroup (31). Patient-reported outcomes (PROs) may also be biased by early treatment discontinuation and by the use of a chronically toxic control arm.

An uncomfortable reality is that IO + TKI combinations are extremely expensive. In high-income settings, costs are enormous but often hidden behind insurance or public systems (32–34). In low- and middle-income countries, and even within constrained healthcare systems in richer nations, universal first-line IO + TKI is simply not feasible (35). Even in big European countries like Spain, access remains an issue in this scenario with no public reimbursement for any of the IO + TKI combos (36). Favorable-risk patients are precisely the group in whom de-escalation could yield the greatest economic and QoL dividends without sacrificing survival in selected patients. Designing and conducting randomized trials of AS versus early IO + TKI, or TKI versus IO + TKI in favorable-risk populations could provide the evidence base to support more sustainable global practice. Until then, insisting that every favorable-risk patient receive IO + TKI as a “standard” risks widening inequities.

The success of IO + TKI combinations in advanced ccRCC is real and transformative, but success in one setting does not justify indiscriminate expansion into all risk groups and biological contexts. In the absence of robust predictive biomarkers, these above considerations support a more thoughtful, efficient and selective use of first-line IO + TKI combinations in favorable-risk ccRCC (37). Combination therapy appears particularly appropriate for patients with high-volume disease, clinically relevant symptoms, or metastatic involvement at risk of rapid progression or organ compromise. In contrast, selected patients with low-volume and asymptomatic disease may be effectively managed with single-agent TKI therapy or AS, allowing for durable disease control while preserving the opportunity for meaningful benefit from immunotherapy in subsequent treatment lines. IO + IO is a valuable alternative in patients that values the possibility of deep and durable responses, at the expense of lower response rates and the risk of immune adverse events.
